# Tracking pregnant women’s mental health through social media: an analysis of reddit posts

**DOI:** 10.1093/jamiaopen/ooad094

**Published:** 2023-11-28

**Authors:** Abhishek Dhankar, Alan Katz

**Affiliations:** Department of Community Health Sciences, Manitoba Centre for Health Policy, Rady Faculty of Health Sciences, University of Manitoba, Winnipeg, MB R3E 3P5, Canada; Department of Family Medicine, Rady Faculty of Health Sciences, University of Manitoba, Winnipeg, MB R3E 0W2, Canada; Department of Community Health Sciences, Manitoba Centre for Health Policy, Rady Faculty of Health Sciences, University of Manitoba, Winnipeg, MB R3E 3P5, Canada; Department of Family Medicine, Rady Faculty of Health Sciences, University of Manitoba, Winnipeg, MB R3E 0W2, Canada

**Keywords:** natural language processing, pregnant women, Reddit, mental health

## Abstract

**Objectives:**

Present an artificial intelligence-enabled pipeline for estimating the prevalence of depression and general anxiety among pregnant women using texts from their social media posts. Use said pipeline to analyze mental health trends on subreddits frequented by pregnant women and report on interesting insights that could be helpful for policy-makers, clinicians, etc.

**Materials and methods:**

We used pretrained transformer-based models to build a natural language processing pipeline that can automatically detect depressed pregnant women on social media and carry out topic modeling to detect their concerns.

**Results:**

We detected depressed posts by pregnant women on Reddit and validated the performance of the depression classification model by carrying out topic modeling to reveal that depressive topics were detected. The proportion of potentially depressed surprisingly reduced during the pandemic (2020 and 2021). Queries related to antidepressants, such as Zoloft, and potential ways of managing mental health dominated discourse before the pandemic (2018 and 2019), whereas queries about pelvic pain and associated stress dominated the discourse during the pandemic.

**Discussion and Conclusion:**

Supportive online communities could be a factor in alleviating stress related to the pandemic, hence the reduction in the proportion of depressed users during the pandemic. Stress during the pandemic has been associated with pelvic pain among pregnant women, and this trend is confirmed through topic modeling of depressive posts during the pandemic.

## Introduction

The COVID-19 pandemic has severely impacted the mental health of various vulnerable populations,^1^ including pregnant women. Tracking the social media posts of pregnant women provides a simple, yet effective method of understanding the effect of COVID-19 on their mental health. Social media sites like Reddit^2^ usually provide a degree of anonymity which helps participants in sharing details about their mental health that they may be more hesitant in revealing in-person. Such messages are in the form of free text that can be analyzed using the latest advancements in natural language processing (NLP, a branch of artificial intelligence [AI] that deals with unstructured textual data) to determine the incidence of mental disorders such as depression and its underlying causes. Such analysis of large-scale data could be helpful to health policy-makers, clinicians, etc., in providing improved care to vulnerable populations, as pregnant women. To achieve that, we collected posts from pregnancy-related subreddits, such as “r/pregnant,” “r/BabyBumps,” “r/CoronaBumpers,” “r/PregnancyUK,” “r/BabyBumpsCanada,” which are sub-forums within Reddit that cater to the interests of the pregnant population.

The contributions of this study are as follows:

- Present an AI-enabled pipeline for estimating the prevalence of depression and general anxiety among pregnant women using only the textual content of their social media posts. The code and associated data have been provided on GitHub^3^ and Open Science Framework Data Repository.^4^
- Use said pipeline to analyze mental health trends on sub-reddits frequented by pregnant women and report on interesting insights that could be helpful for policy-makers, clinicians, etc.

## Background

Concerns have been raised about the need to pay closer attention to the mental health condition of pregnant women,^5^ especially during a pandemic. Prior studies have shown that pregnant women are at especially high risk for developing mental health problems during the pandemic.^6^^,^^7^ In fact, the prevalence of clinically elevated depression and anxiety symptoms had shown a marked increase during the pandemic in comparison to before it (as shown in Fig. 1 in Ref. [8]). Other studies showing an increase in the prevalence of anxiety and depression symptoms during the pandemic include Filippetti et al^9^ and Berthelot et al.^10^ Thus, the tracking of mental health is not only necessary for the well-being of pregnant women, but also for their babies, for instance, perinatal mental disorders, including depression and anxiety, have been associated with poor outcomes for the child.^11^^,^^12^ Furthermore, in a meta-analysis, Rogers et al^13^ concluded that depression and anxiety during antenatal and postnatal periods are associated with adverse outcomes for the child such as poor development of cognitive, language, motor skills, etc. Lockdowns are necessary measures that severely restrict mobility and reduce mental well-being. However, Costa-Font et al^14^ opined that lockdowns could have positive effects on mental health due to reduced “workplace stress” and “improved work-life balance.” These benefits may extend beyond lockdowns—remote work, popularized during the pandemic, could provide all the upsides of a lockdown without many of the downsides. It is possible that lockdowns and working from home may have alleviated some of the stress caused during the pandemic due to the familial social support that may be available at home. Online forums such as pregnancy-focused subreddits could be another way of getting social support, which has been shown to alleviate the risk of depression during pregnancy.^15^^,^^16^

**Figure 1.**
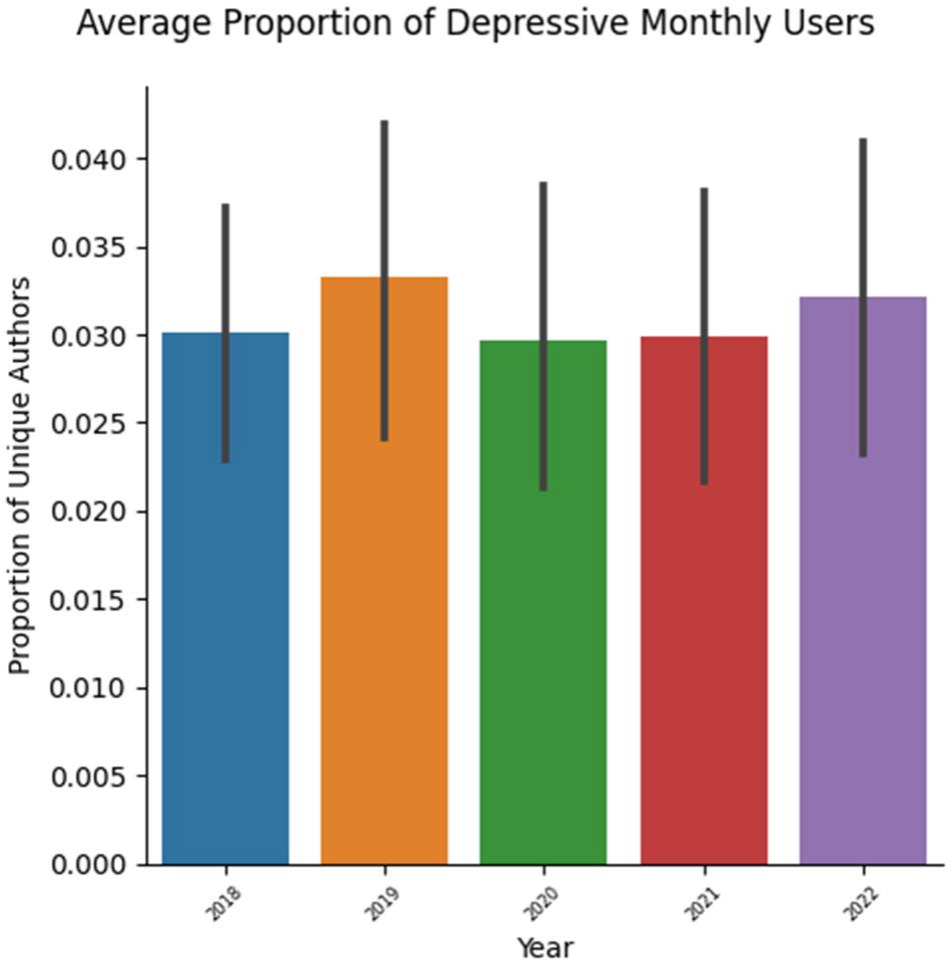
The figure shows the average percentage of unique authors whose posts showed depressive tendencies. The percentage for each month was calculated by dividing the number of unique users posting depressive content by the total number of unique users who posted any content on the subreddit that month.

Tracking the mental health of women through surveys can be costly and time-consuming, and the results could be effected by non-response bias depending on the socioeconomic status of the respondents.^17^ However, the use of social media is prevalent across socioeconomic classes and collection of textual user posts can be easily accomplished at the cost of very little time or money by using internet scrapers, provided the requisite permissions have been taken in advance from the owners of the site.

The analysis of this dataset can be conducted manually. Pilkington et al^18^ carried out a manual content analysis of 217 Reddit posts to determine meaningful themes related to mothers’ worries during pregnancy. Manual curation and analysis of social media comments can be time-consuming and restrict the number of posts analyzed, possibly missing out on important trends in the data. Most automated methods concentrate on detecting changes in mental health support and substance abuse-related subreddits such as “r/Anxiety,” “r/depression,” “r/SuicideWatch,” etc. Low et al^19^ used automated topic modeling called latent Dirichlet allocation (LDA) to analyze the change in topics before and during the pandemic in mental health-related subreddits. Biester et al^20^ examine changes in topics in mental health-related subreddits using LDA and changes in patterns of interactions between users using social network analysis Other studies on automated topic detection also focus on mental health subreddits.^21^^,^^22^ However, none of the aforementioned studies that took a machine learning (ML) and AI-assisted path for the analysis of subreddits focused on pregnant users on Reddit.

A lexical analyzer called LIWC has been usually used to create features to build a classification model.^23^ The issue with LIWC is that it does not take semantic information into consideration. Modern transformers fix that problem, therefore, we will be using a pretrained transformer-based model to track potentially depressive posts on pregnancy-related subreddits.

In a recent Shared Task on Detecting Signs of Depression from Social Media Text (DepSign-LT-EDI@ACL2022), participants were tasked with building a model that could classify social media posts from Reddit into 1 of the 3 classes: “Not Depressed,” “Moderately Depressed,” and “Severely Depressed.”^24^ We use the best-performing model by team OPI for our experiments.^25^ Even though this model was not trained on data specific to pregnancy, it should be able to detect depressive posts in pregnancy-related subreddits because depressive posts tend to share similar linguistics and semantics,^26^^,^^27^ and the model was trained on a dataset containing subreddit posts.

To the best of our knowledge, ours is the first attempt to apply ML and AI techniques to the analysis of pregnancy-related subreddits from a mental health perspective.

## Methods

In this section, we explain the creation of a AL/ML-enabled pipeline. A pipeline is generally a piece of software that carries out a series of operations of the data in order and returns an output. In our case, the pipeline cleans patients’ social media posts (by removing posts according to certain criteria, explained later) and finally applies an ML/AI model to make predictions on the cleaned data to produce an output that tells us about the prevalence of depressive users on pregnancy-related subreddits from 2018 through 2022, as well as about the major topics being talked about before versus during the pandemic.

### Data collection and preprocessing

The data were downloaded using torrent links provided by u/Watchful1.^28^ Specifically, all textual posts (from January 2018 to November 2022) were collected from pregnancy related subreddits, namely, “r/pregnant,” “r/BabyBumps,” “r/CoronaBumpers,” “r/PregnancyUK,” “r/BabyBumpsCanada,” and depression and anxiety related subreddits, namely, “r/SuicideWatch,” “r/depression_help,” “r/depressed,” “r/HealthAnxiety,” “r/AnxietyDepression,” “r/depression,” “r/PanicAttack,” and “r/Anxiety.” The dates were chosen to include data from 2 years before the pandemic, ie, 2018 and 2019, 2 years during the pandemic, ie, 2020 and 2021, and the year 2022 during which many major countries starting transitioning to a post-pandemic normal. At this point, the number of posts under consideration is 320 122.

We then removed all posts that satisfied the following conditions:

- Posts containing the words “discuss,, “daily,” “weekly”—Moderators of subreddits tend to author and open “discussion,” “daily discussion,” and “weekly discussion” posts under which users are allowed to ask and receive answers about their questions without the hassle of going through crafting a new post. These are often questions that are too small to require an independent post and are therefore unlikely to contain details of the posters’ state of mind. Moderators may also open such “discussion threads” to direct users to one place for discussion about major events to prevent multiple users from authoring independent posts asking similar questions.
- Posts by the AutoModerator: The AutoModerator is a bot
- Deleted and Removed posts
- Posts containing fewer than 4 words

After removing the aforementioned posts, we were left with 290 272. While authors on pregnancy-focused subreddits could be assumed to be pregnant women, the same cannot be assumed for mental health subreddits. Therefore, the submissions from the mental health subreddits were filtered by comparing each post to a filtering sentence—“I am pregnant.” This was done through the use of Sentence Transformers, which are pretrained models that can determine if a pair of sentences agree with each other. Only those posts that agreed with the filtering sentence were assumed to be talking about pregnancy, and authors of such posts were assumed to be depressed.

### Depression prediction and topic modeling

The winning model at DepSign-LT-EDI@ACL2022 was used to classify each post in the pregnancy-related subreddits into one of the following classes: “Not Depressed,” “Moderately,” and “Severely” depressed. We merged Moderately and Severely depressed classes into a single class called “Depressed” for further analysis. The total number of “Depressed” posts, thus identified, were 24 383.

Thereafter, we conducted topic modeling on these 24 383 using BerTopic, which is a BERT^29^-based topic modeler. The topic modeling is conducted separately for posts from the years before (2018 and 2019) and during (2020 and 2021) the pandemic.

Our objective now is to count the number of unique authors in every month. To do that, we divided these posts by the year and month of their creation. It is possible that the same author posts multiple times on the subreddit in a single month, thus, to avoid counting duplicates we only count the total number of unique users. The unique users are determined by their unique account ID. While it is possible that 1 author may use multiple accounts to post questions, we cannot uniquely determine the identity of the actual human behind each account due to Reddit’s privacy policies.

## Results

**Figure 2.**
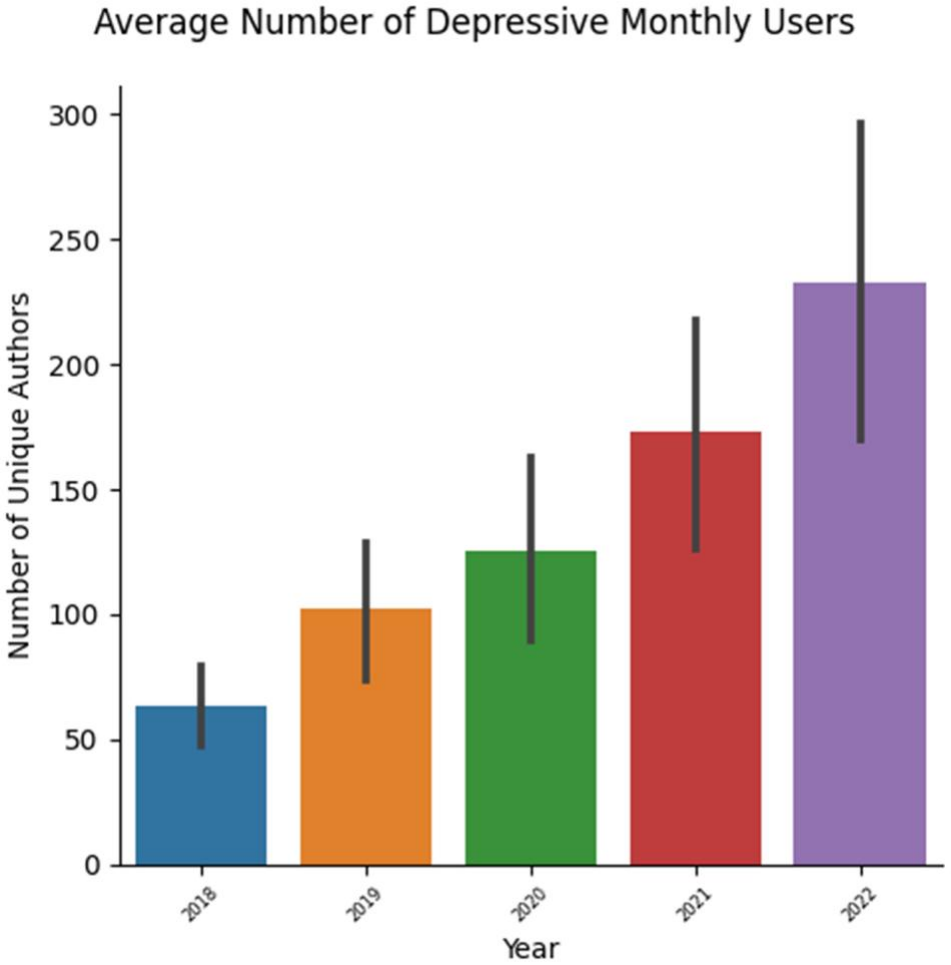
The figure shows the average number of unique authors whose posts showed depressive tendencies.

The total number of textual posts retrieved from 2018 through 2022 from the 13 aforementioned subreddits was 320 122. Of these, 24 383 posts were determined to have depressive content. The results of the depression classification have been plotted in Figures 1 and 2. Figure 1 plots the year in which the author made a comment on the *x*-axis. The bars with the error plots represent the average number of depressive monthly users. These values were calculated by first determining the unique authors that posted depressive content for every class label (refer to DepSign-LT-EDI@ACL2022 in the Background section) in every month of every year being considered. Then the average number of unique authors that posted depressive content each month in a year was plotted in Figure 1. Thereafter, the number of unique authors who posted depressive content each month was divided by the number of unique authors who had posted any content for that month. This value was then plotted in Figure 2.

Lastly, some of the results of the topic modelling on user posts using BerTopic are as follows. Top 5 topics of discussion, in decreasing order of frequency, before the pandemic are as follows:

- taking, Zoloft, anxiety, medication, pregnancy,
- depression, feel, just, like, pregnancy,
- miscarriage, period, pregnant, test, ultrasound,
- weight, body, gained, gain, eating, and
- don, abortion, want, pregnant, baby.

The top 5 topics, in decreasing order of frequency, detected during the pandemic are as follows:

- pain, walk, lower, pelvic, weeks,
- want, abortion, baby, don, child,
- weight, gain, body, gained, eating,
- Zoloft, antidepressants, taking, pregnancy, medication, and
- nausea, eat, water, sickness, ginger.

## Discussion

While Figure 1 shows that the absolute number of authors/users who are showing signs of depression has increased substantially from 2018 to 2022—this can be chalked up to the increase in popularity of Reddit, especially over the pandemic. Figure 2 shows the proportion of authors/users posting depressive content. This reveals that the proportion of depressive users actually declined during the pandemic years (2020 and 2021). However, this is not supported by some of the available literature.^8–10^ Other studies like Smorti et al^30^ point out that hospitalized pregnant women during the pandemic showed no significant increase in depression (vis-a-vis before the pandemic) despite being isolated from their families. Whereas, their counterparts who were not hospitalized saw a significant increase in depression during the pandemic. Additionally, Clifton et al^31^ observed a reduction in the odds of pregnant women experiencing severe anxiety during a lockdown vs pre-lockdown, and anxiety levels following a lockdown were comparable to those prelockdown. It is possible that users joining these new online communities found adequate support and information in these communities from other perinatal women that alleviates anxiety and depression. Smorti et al^30^ also conclude that pregnant and hospitalized women seemed to find social support from other pregnant women. Furthermore, as stated in the Background section, staying close to family during the lockdown and while working from home may have provided some pregnant women with the necessary social support to alleviate anxiety and depression.

The topics detected before and during the pandemic clearly show the discussion of depressive topics and aid in validating the idea that models trained to detect depression in the DepSign-LT-EDI@ACL2022 shared task are able to effectively detect depressive content in pregnancy-related subreddits.

Furthermore, the change in the top 5 topics shows the impact of the COVID-19 pandemic on the discourse in the subreddits. Of particular interest is the appearance of discussion related to pelvic pain, which became the most frequently discussed topic during the pandemic as opposed to discussion about antidepressant medication, such as Zoloft, before the pandemic. Women tend to be at greater risk for pelvic pain than men, and stress has been associated with pelvic pain during the pandemic in pregnant populations,^32^^,^^33^ especially during pregnancy and stressful situations such as a pandemic.

Caution must be taken while interpreting the results of this study because we are assessing the mental state of a human being through indirect means, which can lead to inaccurate conclusions. Since the subjects’ complete demographic and socio-economic information is not available, we cannot correct those biases in our analysis. Lastly, the model itself can make mistakes in classification and mislead the analysis. Further studies on other social media sites and pregnancy-related forums are required to confirm our findings.

## Conclusion

In this study, we presented a novel implementation of existing pretrained models in an NLP pipeline to automatically detect potentially depressed users on subreddits related to pregnancy and to detect posts by pregnant women on mental health-related subreddits. We then presented the results of topic modeling on the corpus of posts classified as depressive posts that validated the performance of the depressive text classification model. Furthermore, our pipeline was able to discover topic trends specific to the COVID-19 pandemic (see pelvic pain in the Discussion section).

This automated method can provide cost and time-effective analysis of emerging concerns of potentially depressed pregnant populations during a health crisis. For example, early detection of emerging issues (eg, the pelvic pain issue or stress due to weight gain) highlighted in our topic modeling could help healthcare professionals in looking out for such stressors among their patients and help in improving their quality of care by treating or suggesting resources to deal with said issues.

Since this is a brief communication, we have plans for future work in this field. We plan on creating a publicly available Python API which will implement the NLP pipeline described in the Methods section. Many of the users provide information about which trimester they are in—this information could be leveraged to provide fine-grained analysis. Instead of combining the Moderate and Severe classes of depression, we could further analyze the posts belonging to each depression category. Furthermore, we could detect mentions of various medical specialists and conduct sentiment analysis to determine the level of satisfaction of pregnant women with respect to different specialists.

## Data Availability

The data are available on the Open Science Framework Data Repository^4^ and the code is available on GitHub.^3^
